# Phenotyping for QTL identification: A case study of resistance to *Plasmopara viticola* and *Erysiphe necator* in grapevine

**DOI:** 10.3389/fpls.2022.930954

**Published:** 2022-08-11

**Authors:** Tyrone Possamai, Sabine Wiedemann-Merdinoglu

**Affiliations:** ^1^CREA—Research Centre for Viticulture and Enology, Conegliano, Italy; ^2^INRAE Centre Grand Est-Colmar, UMR 1131, Colmar, France

**Keywords:** *Erysiphe necator*, grapevine, phenotyping, *Plasmopara viticola*, QTL, resistance breeding, *Rpv*, *Ren/Run*

## Abstract

*Vitis vinifera* is the most widely cultivated grapevine species. It is highly susceptible to *Plasmopara viticola* and *Erysiphe necator*, the causal agents of downy mildew (DM) and powdery mildew (PM), respectively. Current strategies to control DM and PM mainly rely on agrochemical applications that are potentially harmful to humans and the environment. Breeding for resistance to DM and PM in wine grape cultivars by introgressing resistance loci from wild *Vitis* spp. is a complementary and more sustainable solution to manage these two diseases. During the last two decades, 33 loci of resistance to *P. viticola* (*Rpv*) and 15 loci of resistance to *E. necator* (*Ren* and *Run*) have been identified. Phenotyping is salient for QTL characterization and understanding the genetic basis of resistant traits. However, phenotyping remains a major bottleneck for research on *Rpv* and *Ren/Run* loci and disease resistance evaluation. A thorough analysis of the literature on phenotyping methods used for DM and PM resistance evaluation highlighted phenotyping performed in the vineyard, greenhouse or laboratory with major sources of variation, such as environmental conditions, plant material (organ physiology and age), pathogen inoculum (genetic and origin), pathogen inoculation (natural or controlled), and disease assessment method (date, frequency, and method of scoring). All these factors affect resistance assessment and the quality of phenotyping data. We argue that the use of new technologies for disease symptom assessment, and the production and adoption of standardized experimental guidelines should enhance the accuracy and reliability of phenotyping data. This should contribute to a better replicability of resistance evaluation outputs, facilitate QTL identification, and contribute to streamline disease resistance breeding programs.

## Introduction

Grapevine species of the genus *Vitis* display susceptibility, partial, or total resistance to *Plasmopara viticola* Berl. & De Toni, the causal agent of downy mildew (DM), and to *Erysiphe necator* Sch., the causal agent of powdery mildew (PM). *Vitis vinifera* L. is the most cultivated grapevine species worldwide, but most of its accessions are highly susceptible to both *P. viticola* and *E. necator*. DM and PM have devastating impacts on grapevine cultivation (Gessler et al., 2011; Gadoury et al., 2012). Long-lasting research has focused on strategies to control the pathogens of these two diseases (Pertot et al., 2017), including the introgression of resistance from wild grapevine accessions into elite wine grape cultivars (Töpfer et al., 2011; Merdinoglu et al., 2018; Yobrégat, 2018; Töpfer and Trapp, 2022).

The oomycete *P. viticola* and the ascomycete *E. necator* (synonym *Uncinula necator* Burr.) are obligate, biotrophic and polycyclic pathogens, affecting all green organs of their host (Gessler et al., 2011; Gadoury et al., 2012). *Plasmopara viticola* grows optimally under high relative humidity (RH) and mild temperatures (Caffi et al., 2013; Mouafo-Tchinda et al., 2021), while *E. necator* has an optimal growth at 85% RH and 26°C (Carroll and Wilcox, 2003). Under ideal conditions, pathogens develop several cycles of clonal reproduction, causing severe damage to fruit quality and yield. The application of fungicides is commonly used to mitigate the impact of DM and PM in the vineyard, although these agrochemicals can be potentially harmful to humans and the environment (Komárek et al., 2010; Nicolopoulou-Stamati et al., 2016).

American and Asian *Vitis* spp. are frequently studied for disease resistance traits (Staudt and Kassemeyer, 1995; Staudt, 1997; Cadle-Davidson, 2008; Cadle-Davidson et al., 2011) (Tables 1, 2). Most wild grape accessions exhibit partial resistance to *P. viticola* and *E. necator*, affecting several stages of the pathogen life cycle, including the infection frequency, rate of tissue colonization, duration of the latent period, and production of spores without halting the infection (Parlevliet, 1979). A very few *Vitis* species display total resistance to *P. viticola* and *E. necator* with limited pathogen development and an incomplete pathogen life cycle. Research on new germplasms has recently identified resistant *V. vinifera* cultivars with partial resistance to DM and PM in comparison with other *Vitis* spp. (Hoffmann et al., 2008; Coleman et al., 2009; Riaz et al., 2020; Sargolzaei et al., 2020; Possamai et al., 2021).

**Table 1:** Loci of resistance to *P. viticola* (*Rpv*) identified in grapevine. Resistance loci are listed with information about the grapevine accession/species of origin, the putative level of resistance associated with the locus and the environment of phenotyping utilized in the mapping study.

**Locus**	**LG**	**Origin of resistance-accession and species**	**Resistance level^a^**	**Phenotyping environment**	**References**
*Rpv1*	12	“28-8-78” - BC of *V. rotundifolia* “Dearing” (North America)	Partial	Greenhouse	Merdinoglu et al., 2003
*Rpv2*	18	“8624” - BC of *V. rotundifolia*	Total	Laboratory	Wiedemann-Merdinoglu et al., 2006
*Rpv3.1*	18	Multiple interspecific hybrids - *V. rupestris* (North America)	Partial	Field	Fischer et al., 2004; Welter et al., 2007; Bellin et al., 2009; Venuti et al., 2013
				Laboratory	Bellin et al., 2009; Venuti et al., 2013; van Heerden et al., 2014; Zyprian et al., 2016
*Rpv3.2*	18	“Gf.Ga-47-42” interspecific hybrid - *V. rupestris or V. lincecumii* (North America)	Partial weak	Laboratory	Zyprian et al., 2016
*Rpv3.3*	18	“Merzling” interspecific hybrid - *V. riparia* or *V. labrusca* (North America)	Partial weak	Greenhouse Laboratory	Vezzulli et al., 2019
*Rpv4*	4	“Regent” interspecific hybrid – North American *Vitis* spp.	Limited	Field	Welter et al., 2007
*Rpv5* *Rpv6*	9 12	*V.riparia “*Gloire de Montpellier” (North America)	Partial weak Partial weak	Laboratory	Marguerit et al., 2009
*Rpv7*	7	MD	Limited	Laboratory	Bellin et al., 2009
*Rpv8*	14	*V. amurensis* (East Asia)	Partial high	Laboratory	Blasi et al., 2011
*Rpv9* *Rpv13*	7 12	*V.riparia* “Wr 63”	Limited Limited	Field	Moreira et al., 2011
*Rpv10*	9	“Solaris” interspecific hybrid - *V. amurensis*	Partial	Laboratory	Schwander et al., 2012
*Rpv11*	5	MD	Limited	Field	Fischer et al., 2004
				Laboratory	Bellin et al., 2009; Schwander et al., 2012
*Rpv12*	14	“99-1-48” and “Kozma 20/3” interspecific hybrids - *V. amurensis*	Partial high	Field Laboratory	Venuti et al., 2013
*Rpv14*	5	“Börner” interspecific hybrid - *V. cinerea* “Arnold” (North America)	Limited	Field Laboratory	Ochssner et al., 2016
*Rpv17* *Rpv18* *Rpv20* *Rpv21*	8 11 6 7	“Horizon” interspecific hybrid – North American *Vitis* spp.	Limited Limited Limited Limited	Laboratory	Divilov et al., 2018
*Rpv19*	14	*V. rupestris* “B38”	Limited		
*Rpv22* *Rpv23* *Rpv24*	2 15 18	*V. amurensis* “Shuanghong”	Partial weak Partial weak Partial weak	Laboratory	Fu et al., 2020
*Rpv25* *Rpv26*	15 15	*V. amurensis* “Shuangyou”	Partial weak Partial	Laboratory	Lin et al., 2019
*Rpv27*	18	*V. aestivalis* “Norton” (North-America)	Partial weak	Field Laboratory	Sapkota et al., 2019
*Rpv28*	10	*V. rupestris* “B38”	Partial	Greenhouse Laboratory	Bhattarai et al., 2021
*Rpv29* *Rpv30* *Rpv31*	14 3 16	*V. vinifera* “Mgaloblishvili” (Caucasus)	Limited Limited Limited	Laboratory	Sargolzaei et al., 2020

a Resistance level: Total = no pathogen sporulation occurs, explained phenotypic variance is higher than 50%; Partial high = little pathogen development and sporulation occur, explained phenotypic variance is higher than 50%; Partial = pathogen growth and sporulation are delayed and reduced, explained phenotypic variance usually range between 40% and 60%; Partial weak = pathogen growth and sporulation are limitedly delayed and reduced, explained phenotypic variance usually range between 20% and 40%; Limited = resistance conferred by minor-moderate QTL and with little control, possibly not constant, on the trait, explained phenotypic variance is lower than 20%.

Abbreviations: BC, backcross; MD, missing data; LG, linkage group.

**Table 2:** Loci of resistance to *E. necator* (*Ren and Run*) identified in grapevine. Resistance loci are listed with information about the grapevine accession/species of origin, the putative level of resistance associated with the locus and the environment of phenotyping utilized in the mapping study.

**Locus**	**LG**	**Origin of resistance - accession and species**	**Resistance level^a^**	**Phenotyping environment**	**References**
*Ren1*	13	*V. vinifera* “Kishmish vatkana” (Central Asia)	Partial	Greenhouse Field Laboratory	Hoffmann et al., 2008 Cadle-Davidson et al., 2016
*Ren1.2*	13	Multiple *V. vinifera* accessions (Caucasus)	Partial	Greenhouse Laboratory Laboratory	Riaz et al., 2020 Possamai et al., 2021
*Ren2*	14	“Illinois 547-1” interspecific hybrid - *V. cinerea* 'B9' (North America)	Partial weak	Field	Dalbó, 1998; Dalbó et al., 2001
*Ren3*	15	Multiple interspecific hybrids - *V. aestivalis* or *V. rupestris* (North America)	Partial	Field	Welter et al., 2007; Zyprian et al., 2016; Teh et al., 2017
				Greenhouse	van Heerden et al., 2014
				Laboratory	Teh et al., 2017; Zendler et al., 2017, 2021a
*Ren4*	18	Multiple BC of *V. romanetii* (East Asia)	Total	Field	Ramming et al., 2011; Riaz et al., 2011; Mahanil et al., 2012
				Greenhouse Laboratory	Ramming et al., 2011; Mahanil et al., 2012
*Ren5*	14	*V. rotundifolia* “Regale” (North America)	Total	Laboratory	Blanc et al., 2012
*Ren6* *Ren7*	9 19	*V. piasezkii* “DVIT2027” (East Asia)	Total Partial	Field Greenhouse Laboratory	Pap et al., 2016
*Ren8*	18	MD	Limited	Field	Zyprian et al., 2016
*Ren9*	15	“Regent” interspecific hybrid - *V. aestivalis* or *V. rupestris*	Partial	Laboratory	Zendler et al., 2017, 2021a
*Ren10*	2	“MN1264” interspecific hybrid - American *Vitis* spp.	Limited	Field Laboratory	Teh et al., 2017
*Ren11*	15	“Tamiami” - BC of *V. aestivalis* ‘Femmel 6'	Partial	Field	Karn et al., 2021
*Run1*	12	“VRH3082-1-42” - BC of *V. rotundifolia “*G52”	Total	Field Greenhouse Laboratory	Pauquet et al., 2001
*Run2.1*	18	“JB81-107-11” interspecific hybrid *- V. rotundifolia “*Magnolia”	Partial	Field	Riaz et al., 2011
*Run2.2*	18	“e2-9” - BC of *V. rotundifolia* “Tryshed”	Partial	Field	Riaz et al., 2011

a Resistance level: Total = no pathogen sporulation occurs, explained phenotypic variance is higher than 50%; Partial high = little pathogen development and sporulation occur, explained phenotypic variance is higher than 50%; Partial = pathogen growth and sporulation are delayed and reduced, explained phenotypic variance usually range between 40% and 60%; Partial weak = pathogen growth and sporulation are limitedly delayed and reduced, explained phenotypic variance usually range between 20% and 40%; Limited = resistance conferred by minor-moderate QTL and with little control, possibly not constant, on the trait, explained phenotypic variance is lower than 20%.

Abbreviations: BC, backcross; MD, missing data; LG, linkage group.

The genetic basis of resistance traits in grapevine is usually identified through the biparental mapping approach with the construction of genetic maps performed by crossing two heterozygous parents and by separately analyzing their segregating markers in the progenies (pseudo-testcross mapping strategy; Grattapaglia and Sederoff, 1994). Selfing populations are an alternative to this approach (Blasi et al., 2011; Blanc et al., 2012). For quantitative trait loci (QTL) analyses, progenies are divided into groups according to the inherited genotypes and their phenotypes are compared to identify significant associations between traits and allelic variants.

Genetic maps and QTL analyses were influenced early on by the availability and reproducibility of markers. The lack of a genome reference maps created erroneous marker associations with different linkage groups, which made the comparison of studies and the interoperability of the results difficult (Marino et al., 2003; Marguerit et al., 2009). Currently, the use of high throughput sequencing technologies, more robust softwares and hardwares, and the availability of the grape reference genome (Jaillon et al., 2007; Canaguier et al., 2017) facilitate the discovery of thousands of molecular markers, the unification of linkage groups, the verification of the marker collinearity with the reference sequence, and the production of precise genetic maps (Hyma et al., 2015; Cadle-Davidson et al., 2016; Possamai et al., 2021).

To date, more than 50 populations have been genotyped and phenotyped to map QTL of resistance to *P. viticola* and *E. necator*. Mapping populations were generated by crossing *V. vinifera* with either wild *Vitis* spp. (Marguerit et al., 2009; Pap et al., 2016; Lin et al., 2019; Sapkota et al., 2019), backcross individuals (Pauquet et al., 2001; Riaz et al., 2011; Mahanil et al., 2012; Karn et al., 2021), or hybrid accessions (e.g., Welter et al., 2007; Bellin et al., 2009; Venuti et al., 2013; Vezzulli et al., 2019) (Tables 1, 2). Only one genome-wide association study (GWAS) based on both cross-generated individuals and natural grape cultivars was performed (Sargolzaei et al., 2020), and only two studies used pedigree information to characterize resistance QTL (Di Gaspero et al., 2012; Peressotti et al., 2015).

Genetic studies identified 33 loci of resistance to *P. viticola* (*Rpv*), and 15 loci of resistance to *E. necator* (*Ren* and *Run*) in American, Asian *Vitis* spp. and in some *V. vinifera* cultivars (Tables 1, 2) (Merdinoglu et al., 2018; Dry et al., 2019; https://www.vivc.de). Some *Rpv* and *Ren/Run* loci are labeled as major if they explain large phenotypic variance (usually more than 20%; Dalbó, 1998; Pauquet et al., 2001; Merdinoglu et al., 2003; Fischer et al., 2004; Blasi et al., 2011; Riaz et al., 2011, 2020; Blanc et al., 2012; Schwander et al., 2012; van Heerden et al., 2014; Zyprian et al., 2016; Teh et al., 2017; Zendler et al., 2017; Sapkota et al., 2019; Vezzulli et al., 2019; Bhattarai et al., 2021) and are stable across experiments. These loci confer total (Ramming et al., 2011; Blanc et al., 2012; Pap et al., 2016) or partial resistance (Dalbó et al., 2001; Pap et al., 2016; Lin et al., 2019; Possamai et al., 2021) (Tables 1, 2). Other loci are classified as minor or moderate when QTL have a limited control on the trait and pathogen development, usually up to 20% of explained phenotypic variance (Welter et al., 2007; Moreira et al., 2011; Schwander et al., 2012; Teh et al., 2017) and/or are mapped erratically in different replicates of the resistance evaluation (Fischer et al., 2004; Welter et al., 2007; Bellin et al., 2009; van Heerden et al., 2014; Zyprian et al., 2016; Lin et al., 2019) (Tables 1, 2).

In resistance mapping studies and loci characterization, phenotyping remains the main bottleneck. Indeed, sources of variation, such as environmental conditions, plant material, inoculum and inoculation of the pathogen and disease assessment method, affect resistance assessment and the quality of phenotyping data. In this review, we analyze different approaches used for phenotyping DM and PM resistance in grapevine, and discuss factors that contribute to the accuracy and reliability of phenotyping data, while exploring avenues for improvement.

## Phenotyping of *Vitis* spp. resistance to *P. viticola* and *E. necator* in different environments

In the past 20 years, phenotyping strategies to map *Rpv* and *Ren/Run* loci showed a variability of protocols employed in different environments (vineyard, greenhouse or laboratory). This variability in loci characterization seems to be impacted by the receptivity of the plant material to infection, control of the pathogen inoculum, management of experimental conditions, and resistance variable assessed.

Vineyard assessments have been preferred for studying grapevine resistance to *E. necator* with 12 *Ren/Run* loci identified, while laboratory (*in vitro*) assays were more frequently used for *P. viticola* evaluations with at least 27 *Rpv* loci identified. Evaluations in the greenhouse have been used to a lower extent with only 3 *Rpv* and 7 *Ren/Run* loci characterized (Tables 1, 2).

## Phenotyping in the vineyard

Resistance phenotyping in the vineyard has been performed since the 19th century (Töpfer et al., 2011; Yobrégat, 2018; Töpfer and Trapp, 2022). Candidate vines in a vineyard are assessed starting in the 3rd-year post-planting. Vines are maintained with or without a minimal fungicide protection, and scored for 2 (most of the studies) to 9 years (Karn et al., 2021). This approach enables the evaluation of resistance at the whole plant level under natural environmental conditions in the context of natural pathogen populations (that may vary over years) and polycyclic infections.

### Plant material receptivity

Disease resistance in plants is genetically defined but, in some cases, is organ-specific (Hermanns et al., 2003; Strugala et al., 2015). For grapevine, cases with large differences in the level of resistance between organs (leaves and bunches) are quite rare and seem to apply to only a few accessions (Kennelly et al., 2005; Buonassisi et al., 2018). Leaves are the most rated organ for disease resistance, probably because they are numerous, available throughout the vegetative season, bidimensional, and can be scored for different variables. Resistance to DM and PM may also be assessed on berries, rachises, and canes in the vineyard (Riaz et al., 2011; Pap et al., 2016; Karn et al., 2021).

Environmental conditions may cause host stress-induced resistance and reduced DM and PM infections. For instance, temperatures below 8°C (Moyer et al., 2010), nutrient deficiency (Keller et al., 2003), and drought (Heyman et al., 2021) reduce the development and susceptibility of grapevine organs to diseases, delaying epidemics (Moyer et al., 2016). In addition, cultural strategies (soil tillage and fertilization) and stimulated grapevine growth increase both leaf and berry susceptibility to pathogens (Fernandes de Oliveira et al., 2021).

For grapevines growing under optimal conditions, resistance phenotypes are affected by the development stage of the organ, its physiology, and source-sink relationships. Young tissue shows higher susceptibility to DM and PM with the exception of very early leaves to *P. viticola* because of low stomata receptivity (Kennelly et al., 2007). Old grapevine tissue shows ontogenic resistance (Delp, 1954; Kennelly et al., 2005; Develey-Rivière and Galiana, 2007). For instance, leaves are less susceptible to *E. necator* after their physiological transition from sink to source status (Doster and Schnathorst, 1985; Calonnec et al., 2018). They are also characterized by an overexpression of pathogenesis-related (PR) protein genes, and ethylene and phenylpropanoid pathways (Calonnec et al., 2021). The ontogenic resistance process is promoted by stress and appears to be cultivar-dependent (Doster and Schnathorst, 1985; Barba et al., 2015; Calonnec et al., 2018, 2021). In particular, the gain in resistance is considered higher in partially resistant cultivars (Peros et al., 2006). Grape berries and rachises are receptive to DM until stomata convert to lenticels, 4–6 weeks post-bloom (Kennelly et al., 2005) and are receptive to PM until they reach 8% sugar content (Delp, 1954; Gadoury et al., 2003).

### Evolution of pathogen populations and isolates in natural environment

Pathogen populations in the vineyard are characterized by some genetic diversity and undergo evolutionary processes with varied selection pressures. *Plasmopara viticola* and *Erysiphe necator* originated in North America where the genetic variability is high (Brewer and Milgroom, 2010; Rouxel et al., 2013). For instance, cryptic species of *P. viticola* are described on different *Vitis* spp. (Rouxel et al., 2013; Taylor et al., 2019) and specialized isolates of *E. necator* are detected on *V. rotundifolia* (Frenkel et al., 2010). In Europe, Australia, and other countries, pathogen diversity is lower due to the bottleneck at the time of their introduction (Brewer and Milgroom, 2010; Fontaine et al., 2013, 2021; Taylor et al., 2019). In Europe, two dominant *E. necator* isolates termed EU-A and B have been characterized (Délye et al., 1997; Brewer and Milgroom, 2010; Csikós et al., 2020). Group A populations are clonal while group B populations are sexual, but these two groups differ little in aggressiveness (Peros et al., 2006).

Distinct populations of *P. viticola* and *E. necator* may be involved in divergent resistance phenotyping outputs and mapping studies (Cadle-Davidson, 2008; Cadle-Davidson et al., 2016). Selective pressures, such as fungicide treatments (Frenkel et al., 2015; Delmas et al., 2017; Santos et al., 2020; Massi et al., 2021) and host resistance, are responsible for changes in population biology over time. In Europe, extensive asexual reproduction and mutation rates of *P. viticola*, despite little genetic differentiation, yield virulent isolates on *Rpv3.1* (Peressotti et al., 2010; Venuti et al., 2013; Delmotte et al., 2014), *Rpv10* (Heyman et al., 2021), and *Rpv12* (Wingerter et al., 2021) grapevine genotypes. Similarly, phenotyping data in North America suggest the selection of natural virulent *E. necator* isolates on *Ren2* (Cadle-Davidson et al., 2016) and *Run1* (Feechan et al., 2015) grapevine genotypes.

Additionally, DM and PM inoculum pressure and distribution vary with geographic location, vineyard, and season in any given year (Montarry et al., 2008; Boso et al., 2019). Extremely low humidity and low or high temperatures are the main factors that negatively affect *P. viticola* and *E. necator* growth and sporulation (Caffi et al., 2013; Choudhury et al., 2014). Assessments for resistance are conducted one to three times annually. However, the end of the vegetative season, when the damage of DM and PM is usually more intense, is the preferred period for scoring resistance with dates changing depending on the year (Ramming et al., 2011; Riaz et al., 2011; Pap et al., 2016; Zyprian et al., 2016; Teh et al., 2017; Zendler et al., 2017; Karn et al., 2021).

### Resistance traits can be measured by several variables and scales

Resistance phenotypic variables are scored by visually monitoring disease symptoms that are determined by the plant response to inoculation (necrosis) and/or pathogen growth. In the vineyard, *P. viticola* early infections cause yellow lesions on leaves that appear as translucent oil spots. On older leaves in late season, infections are limited and form yellow to reddish-brown spots in a mosaic-like patchwork. The sporulation of *P. viticola* is white, downy, and cottony, and develops on the abaxial leaf surface. The infection of *E. necator* displays on green tissues as whitish and powdery colonies that get thicker and larger with pathogen mycelium growth and sporulation. Numerous brown to black necrosis can be present during both DM and PM infections. Young berries infected by DM produce sporulation or shrivel completely in later infections, while in the case of PM infections, can break off during growth.

DM and PM symptoms in the vineyard are evaluated on one to three plants per genotype by determining the number of infected leaves (disease incidence) or by different visual scales (disease severity) (Table 3). The use of standardized descriptors determined by the Organization Internationale de la Vigne et du Vin (OIV; OIV, 2009) or the International Plant Genetic Resources Institute (IPGRI) represents a solid foundation for cross-comparison of results (Cadle-Davidson et al., 2019). The OIV codes 452 and 455 for leaf resistance, and 453 and 456 for berry resistance to *P. viticola* and *E. necator*, respectively, refer to a semi-quantitative scoring method. The scales comprise five classes, ranging from 1 for very susceptible phenotypes (with extended and dense pathogen sporulation) to 9 for totally resistant phenotypes (with only small necrosis or no symptoms) (OIV, 2009) (Table 3). Inverted scales from IPGRI positively correlated with the rate of infection are frequently considered to reduce the risk of possible confusion between the rate of disease severity and the degree of plant resistance (Pap et al., 2016; Teh et al., 2017; Zendler et al., 2017). This scale is also applied to various organs (Riaz et al., 2011; Pap et al., 2016; Karn et al., 2021) (Table 3).

**Table 3:** Resistance variables assessed in the vineyard and greenhouse. An abbreviation and a description of the method and scale utilized for the scoring is proposed for each resistance variable assessed in mapping studies.

**Resistance variable ID**	**Trait**	**Method**	**Scale (susceptible to resistant)**
OIV 452; OIV 452i	Leaf degree of resistance to *P. viticola*	Visual semi-quantitative estimation of pathogen infection	Discrete 1 to 9; Discrete 9 to 1 (i)
OIV 453; OIV 453i	Cluster degree of resistance to *P. viticola*	Visual semi-quantitative estimation of pathogen infection	Discrete 1 to 9; Discrete 9 to 1 (i)
DOS	Leaf degree of resistance to *P. viticola*	Visual measurement of the diameter of oil spots (DOS)	Centimeters
OIV 455; OIV 455i (or IPGRI)	Leaf degree of resistance to *E. necator*	Visual semi-quantitative estimation of pathogen infection	Discrete 1 to 9; Discrete 9 to 1 (i)
OIV 456; OIV 456i	Cluster degree of resistance to *E. necator*	Visual semi-quantitative estimation of pathogen infection	Discrete 1 to 9; Discrete 9 to 1 (i)
DI	Leaf degree of resistance to *P. viticola* or *E. necator*	Disease incidence (DI): visual count or classification of infected plants/organs	Number of counted units
DS	Leaf degree of resistance to *P. viticola* or *E. necator*	Disease severity (DS): visual semi-quantitative estimation of the infection	Discrete: 1 to 5 (Dalbó, 1998); 1 to 4 (Ramming et al., 2011; Karn et al., 2021); 0 to 5 (Riaz et al., 2011; Pap et al., 2016); 1 to 7 (Teh et al., 2017); 1 to 9 (Karn et al., 2021); 1 to 10 (Bhattarai et al., 2021)
NS	Leaf degree of resistance to *P. viticola*	Necrosis severity/size (NS): visual semi-quantitative estimation	Discrete 9 to 1
AUDPC	Leaf degree of resistance to *P. viticola* or *E. necator*	Calculation according to Jeger and Viljanen-Rollinson (2001)	According to the scale of single time point assessment

Abbreviations: AUDPC, Area Under Disease Pressure Curve; i, inverted resistance scale; ID, identity; IPGRI, International Plant Genetic Resources Institute; OIV, Organisation Internationale de la Vigne et du Vin.

### QTL identification in vineyard conditions: Stability over time, sites, and organs

Phenotyping in the vineyard uncovered the following major resistance QTL: *Rpv3.1* (Welter et al., 2007; Bellin et al., 2009; Zyprian et al., 2016), *Rpv12* (Venuti et al., 2013), *Rpv14* (Ochssner et al., 2016), *Rpv27* (Sapkota et al., 2019), *Ren1* (Cadle-Davidson et al., 2016), *Ren2* (Dalbó, 1998; Dalbó et al., 2001; Cadle-Davidson et al., 2016), *Ren3/Ren9* (Fischer et al., 2004; Welter et al., 2007; Zyprian et al., 2016; Teh et al., 2017; Zendler et al., 2017), *Ren4* (Ramming et al., 2011), *Ren6, Ren7* (Pap et al., 2016), *Ren11* (Karn et al., 2021), and *Run2* (Riaz et al., 2011) (Table 4). Some of these resistance QTLs such as *Rpv3.1* (Welter et al., 2007; Zyprian et al., 2016) and *Ren3/Ren9* (Fischer et al., 2004; Welter et al., 2007; Teh et al., 2017) are stable over time and across studies. *Ren3 and Ren9* are two close QTLs often detected as one locus, whose position is shifted during annual evaluations, probably due to the prevalence of different PM isolates from the beginning to the end of the epidemic (Zendler et al., 2017, 2021a). Moderate or minor QTL were difficult to detect because of the effects of major QTL, such as for *Ren10* (Teh et al., 2017), or because of unique climatic conditions, such as for *Ren8* (Zyprian et al., 2016), or because of their low and unstable significance, such as for *Rpv4* and *Rpv11* (Fischer et al., 2004; Welter et al., 2007). Disease resistance QTL do not appear to be organ-specific in grapevine, but show different significance and explained phenotypic variance depending on the organ considered: *Rpv3.1* and *Ren3/Ren9* were identified on leaves and berries (Welter et al., 2007; Zyprian et al., 2016); and *Ren11* (Karn et al., 2021) and *Run2.1* (Riaz et al., 2011) on leaves, berries, rachises, and canes; *Ren6* and *Ren7* were detected on leaves and canes (Pap et al., 2016); and the minor QTL *Ren8* was associated with resistance in both leaves and berries (Zyprian et al., 2016), while *Rpv4* was identified only with leaf resistance variables (Welter et al., 2007). Larger and more constant QTL significance is detected for leaf phenotypes while berry susceptibility seems more affected by year or environmental conditions (Vezzulli et al., 2018) and may depend on ideal infection conditions, during the limited time of berry receptivity.

**Table 4:** Resistance phenotyping strategies utilized in the vineyard. The main elements and possible sources of variation of phenotyping protocols adopted in the main mapping studies are reported for each *Rpv* and *Ren/Run* locus.

**Locus**	**Plant material - observation unit**	**Pathogen inoculum**	**Resistance variables ID**	**Experimental design**			
				**Unit NB**	**Time of observations**	**Replicates**	**References**
*Rpv3.1; Rpv4; Rpv11; Ren3*	Whole plant - Leaves and clusters separately	Natural	OIV 452i; OIV 453i; NS; OIV 455i; OIV 456i	1	MD	Up to 5 years	Fischer et al., 2004; Welter et al., 2007
*Rpv3.1*	Whole plant	Natural	DI	2	Daily for MD days	2 years	Bellin et al., 2009
*Rpv3.2; Ren3; Ren8*	Whole plant - Leaves and clusters separately	Natural	OIV 452i; OIV 453i; OIV 455i; OIV 456i	2 or 3	Once - ES	Up to 6 years	Zyprian et al., 2016
*Rpv9; Rpv13*	Shoots	Controlled by spraying	DS; DOS	3	15 dpi	3 years	Moreira et al., 2011
*Rpv12*	Whole plant	Natural	OIV 452	MD	MD	2 years	Venuti et al., 2013
*Rpv14*	Whole plant	Natural	OIV 452i	2	3 times	4 years	Ochssner et al., 2016
*Rpv27*	Whole plant	Natural	OIV 452i	MD	3 times	2 years	Sapkota et al., 2019
*Ren1; Ren2*	Whole plant - 2^nd^-3^rd^ leaf, rachis, berries and stems	Natural	DS; IPGRI	1	One or two - ES	Up to 4 years	Cadle-Davidson et al., 2016
*Ren2*	Whole plant	Natural	DS	1	Once - ES	5 years	Dalbó, 1998; Dalbó et al., 2001
*Ren3; Ren9*	Whole plant	Natural	OIV 455i	1 or 2	Up to 4 times - ES	Up to 8 years	Zendler et al., 2017, 2021a
*Ren3; Ren10*	Whole plant	Natural	DS; IPGRI; AUDPC	1	Once - ES; 8-10 times for AUDPC	2 years	Teh et al., 2017
*Ren4*	Whole plant	Natural	DS	2	MD times - ES	3 years	Ramming et al., 2011
*Ren6; Ren7*	Whole plant - Leaves and canes separately	Natural	DS	1	Once per month per 3 months - ES	1 year	Pap et al., 2016
		Controlled by spraying	DS	1	Once per month per 2 months - ES	1 year	
*Ren11*	Whole plant - Leaves, rachis, berries and stems	Natural	DS; AUDPC	MD	Once or twice - ES; Twice a week for MD weeks for AUDPC	Up to 9 years	Karn et al., 2021
*Run1*	Whole plant	Natural	MD	MD	MD	1 year	Pauquet et al., 2001
*Run2.1; Run2.2; Ren4*	Whole plant - leaves, canes, rachis and berries	Natural	DS	1	Once - ES	Up to 3 years	Riaz et al., 2011

Abbreviations: MD, missing data; ES, end of season; No., number; dpi, days post infection; Resistance variables ID: see Table 3.

## Phenotyping in the greenhouse

As an alternative to vineyard assessment and its variable experimental conditions, the greenhouse is a more controlled environment which enables optimized plant and pathogen development. DM and PM infections appear spontaneously during the season or are induced by artificial inoculation on the whole plant or defined leaves.

### Control of phenotyping sources of variation in the greenhouse

Phenotyping in the greenhouse usually relies on two to four replicates of each genotype obtained from hard-wood or soft-wood cuttings. Plants are maintained for 1–3 years in pots and are fertilized and pruned to maintain young growing shoots during the evaluation period. Plants in the greenhouse may be affected by only minor non-homogeneous conditions such as light exposure (Cadle-Davidson, 2008). As applied in the vineyard, grapevines are maintained in the greenhouse with or without a minimal fungicide protection.

Conditions for *P. viticola* infections in the greenhouse can be natural (Bhattarai et al., 2021) or induced (Merdinoglu et al., 2003). Artificial *P. viticola* inoculations are usually preferred and performed using a natural pathogen population from the vineyard (Moreira et al., 2011), or using a defined isolate maintained on controlled potted plants (Merdinoglu et al., 2003; Bellin et al., 2009; Vezzulli et al., 2019). Sporangia are sprayed by a water solution on receptive leaves of potted plants (Merdinoglu et al., 2003; Bellin et al., 2009) or on whole plants (Vezzulli et al., 2019; Bhattarai et al., 2021). DM is evaluated once annually between 7 (Merdinoglu et al., 2003) and 15 days post-inoculation (dpi) (Moreira et al., 2011) (Table 5).

**Table 5:** Resistance phenotyping strategies utilized in the greenhouse. The main elements and possible sources of variation of phenotyping protocols adopted in the main mapping studies are reported for each *Rpv* and *Ren/Run* locus.

**Locus**	**Plant material - observation unit**	**Pathogen inoculum**			**Experimental conditions**	**Resistance variables ID**	**Experimental design**			
		**Inoculation**	**Origin**	**Way of inoculation**			**Units NB**	**Time of observations**	**Replicates**	**References**
*Rpv1*	Leaves	Controlled	MD	Spray	Inoculation under wet tents	OIV 452	1	7 dpi	1 year	Merdinoglu et al., 2003
*Rpv3.1; Rpv11*	Whole plant	Natural			MD	OIV 452	1	3 times	1 year	Welter et al., 2007
*Rpv3.3*	Leaves	Controlled	Artificially infected plants	Spray 1 x 10^5^ sporangia/ml	25/20°C day/night; 16/8 h light/dark; 70 ± 10% RH	OIV 452	6	8 dpi	2 years	Vezzulli et al., 2019
*Rpv9; Rpv13*	Whole plant	Controlled	Field	Spray 4.25 x 10^5^ sporangia/ml	20°C; 80% RH	DS	3	15 dpi	1 year	Moreira et al., 2011
*Rpv28*	Whole plant	Natural			MD	DS	2	5 dpi after first symptoms development	1 year	Bhattarai et al., 2021
*Ren1*	Whole plant	Partially controlled	Naturally infected plants	Proximity	22–27°C; 72–96% RH	OIV 455	1	Every 3 weeks for 3 times	2 years	Hoffmann et al., 2008
*Ren1.2*	Whole plant	Controlled	Leaves *in vitro*	Spray 7 x 10^4^ conidia/ml	23 to 27°C; 12 h min of light	DS	4	28 dpi	1 year	Riaz et al., 2020
*Ren3*	Whole plant	Controlled	Artificially infected plants	Proximity	MD	OIV 455	1	14 dpi	3 years	van Heerden et al., 2014
*Ren4*	Whole plant	Partially controlled	Naturally infected plants	Proximity	MD	DI; DS	2 or 3	Twice -ES	1 year	Ramming et al., 2011
*Ren6; Ren7*	Whole plant	Controlled	MD	Spray 7 x 10^4^ conidia/ml	23 to 27°C; 12 h min of light	DS	3 or 4	14 dpi	1 year	Pap et al., 2016
*Run1*	Whole plant	Controlled	Field	Sprays once a week for 3 weeks	MD	DI	MD	30 dpi	1 year	Pauquet et al., 2001

Abbreviations: dpi, days-post-inoculation; ES, end of season; MD, missing data; No., number; RH, relative humidity; NB, number; Resistance variables ID: see Table 3.

In the greenhouse, *E. necator* encounters optimal growth conditions and may develop natural infections (De Nardi et al., 2019). However, to encourage PM and its homogeneous distribution, naturally or artificially infected *V. vinifera* plants are often placed among the population of grapevines to be phenotyped (Hoffmann et al., 2008; Ramming et al., 2011; Mahanil et al., 2012; van Heerden et al., 2014; Karn et al., 2021). Then, the epidemic develops over several months and plants are evaluated two or three times (Hoffmann et al., 2008; Ramming et al., 2011; van Heerden et al., 2014; Karn et al., 2021). Alternatively, the pathogen may be inoculated by spraying and resistance is evaluated after a few weeks (Pauquet et al., 2001; Pap et al., 2016).

In the greenhouse, resistance is scored according to the OIV descriptors as done in the vineyard (Merdinoglu et al., 2003; Hoffmann et al., 2008; van Heerden et al., 2014; Vezzulli et al., 2019) or by using other similar semi-quantitative scales (Moreira et al., 2011; Ramming et al., 2011; Bhattarai et al., 2021) (Table 5).

### QTL identification in greenhouse conditions

Greenhouse bioassays are mostly carried out with no or a few experimental replicates. These conditions enabled the identification of *Rpv1* (Merdinoglu et al., 2003), *Rpv3.3* (Vezzulli et al., 2019), *Rpv28* (Bhattarai et al., 2021), *Ren1* (Hoffmann et al., 2008), *Ren1.2* (Riaz et al., 2020), and *Run1* (Pauquet et al., 2001) (Table 5). Phenotyping in the greenhouse also confirmed QTL identified under vineyard conditions. This occurred for *Rpv3.1* (Welter et al., 2007), *Ren3/Ren9* (van Heerden et al., 2014), *Ren4* (Ramming et al., 2011), and *Ren6* and *Ren7* (Pap et al., 2016). Only *Ren11* was not confirmed with greenhouse-collected data (Karn et al., 2021). At the same time, greenhouse assays showed a lower number of minor QTLs, confirming those from the vineyard data, such as *Rpv11* (Welter et al., 2007) or suggested possible new ones (Karn et al., 2021) (Table 5). Multi-year greenhouse assessments showed a very stable QTL identification for *Ren3/Ren9* with data consistently collected at the same time after inoculation (van Heerden et al., 2014). Furthermore, a multi-phenotype study identified the highest QTL significance by visual greenhouse evaluation in comparison with vineyard and *in vitro* assays (Pap et al., 2016). These results suggest the potential of standardized greenhouse phenotyping in the evaluation of grapevine resistance to pathogens.

## Phenotyping under *in vitro* conditions

The evaluation of grapevine resistance in the laboratory or *in vitro* conditions requires limited resources and space for plants and pathogens, and allows for an increase of genotypes to be evaluated and of bioassay replicates. One or multiple experiments per year can be performed (Schwander et al., 2012; Divilov et al., 2017; Possamai et al., 2021) and can integrate phenotypic dataset from the greenhouse or vineyard (Vezzulli et al., 2019; Riaz et al., 2020; Bhattarai et al., 2021). For these reasons, the *in vitro* phenotyping strategy is considered as an effective and practical tool for screening large populations and for identifying resistant accessions among grapevine collections (Brown et al., 1999; Deglène-Benbrahim et al., 2010; Miclot et al., 2012; Calonnec et al., 2013; Buonassisi et al., 2018; Vezzulli et al., 2018; Possamai et al., 2020). This approach is frequently used for resistance QTL identification.

Some tendencies were observed among the *in vitro* protocols described in the literature. Some mapping studies used different protocols (Pauquet et al., 2001; Bellin et al., 2009; Blanc et al., 2012; Schwander et al., 2012; Pap et al., 2016; Divilov et al., 2017; Sapkota et al., 2019; Vezzulli et al., 2019; Karn et al., 2021) but others used very similar protocols (either Bellin et al., 2009; Marguerit et al., 2009; Blasi et al., 2011; Venuti et al., 2013, or Schwander et al., 2012; Ochssner et al., 2016; Zyprian et al., 2016). The latter reports suggested possible standardizations for *in vitro* phenotyping bioassays.

In the laboratory, resistance is evaluated during a single pathogen life cycle, which happens during a very short time in comparison with epidemics under natural conditions. Under *in vitro* conditions, the origin and preparation of the plant material, the origin of the pathogen isolates, the methods of inoculation, the time point used for rating and the resistance variables assessed represent sources of variation in resistance evaluation that may affect the phenotyping results, although these factors can be managed (Tables 6, 7).

**Table 6:** Resistance variables assessed in the laboratory. An abbreviation and a description of the method and the scale utilized for the scoring are proposed for each resistance variable assessed in mapping studies.

**Variable ID/name**	**Trait**	**Method**	**Scale (susceptible to resistant)**
OIV 452-1; OIV 452-1i; OIV 452a	Leaf degree of resistance to *P. viticola*	Visual semi-quantitative, or automatic image-based quantitative (a), estimation of pathogen infection	Discrete 1 to 9; Discrete 9 to 1 (i);
MYC	Leaf degree of resistance to *P. viticola* or *E. necator*	Visual semi-quantitative estimation of pathogen mycelium growth (MYC)	Discrete 1 to 9
HZ	Leaf degree of resistance to *E. necator*	Visual metric measurement of longest hyphae	Micrometer
SPO	Leaf degree of resistance to *P. viticola* or *E. necator*	Visual semi-quantitative estimation of pathogen sporulation (SPO)	Discrete 1 to 9
HT	Leaf degree of resistance to *P. viticola* or *E. necator*	Hyphal transects (HT) count the number of interceptions of individual hyphae crossing one of two axial transects	Counted transects
SD; SDi	Leaf degree of resistance to *P. viticola*	Visual semi-quantitative estimation of sporangia density (SD)	Discrete 1 to 9; Discrete 9 to 1 (i);
SZ	Leaf degree of resistance to *P. viticola*	Visual metric measurement of sporangia size (SZ)	Micrometer
SCCv; SCCa	Leaf degree of resistance to *P. viticola* or *E. necator*	Sporangia/conidia count (SCC) in water solution by microscope (v) or automatic cell counter (a)	Counted structures
qPCR	Leaf degree of resistance to *E. necator*	*E. necator* mass quantification through real-time PCR	Continuous values starting from 1
DI	Leaf degree of resistance to *P. viticola* or *E. necator*	Disease incidence (DI): visual count of infected leaves/leaf disks	Number or proportion of counted units
DS; DSa	Leaf degree of resistance to *P. viticola or E. necator*	Disease severity (DS): visual semi-quantitative, or automatic image-based quantitative (DSa), estimation of the infection e.g., % of infected surface or amount of hyphae/microcolonies.	0 to 100% (Ramming et al., 2011; Divilov et al., 2017, 2018; Vezzulli et al., 2019; Zendler et al., 2021a). Discrete: 0 to 4 (Pap et al., 2016); 0 to 7 (Sargolzaei et al., 2020); 1 to 4 (Cadle-Davidson et al., 2016); 1 to 5 (Divilov et al., 2017, 2018; Sapkota et al., 2019; Riaz et al., 2020); 1 to 9 (Fu et al., 2020).
NI	Leaf degree of resistance to *P. viticola or E. necator*	Necrosis incidence (NI): count of plant material units with plant necrosis patches/spots	Number or proportion of counted units
NS; NSa	Leaf degree of resistance to *P. viticola or E. necator*	Visual semi-quantitative, or automatic image-based quantitative (NSa), estimation of necrosis extension/size/severity (NS):	Discrete: 0–3 (Zendler et al., 2021a); 1–5 (Divilov et al., 2017, 2018); 1–9 (Zyprian et al., 2016);
NF	Leaf degree of resistance to *P. viticola or E. necator*	Visual semi-quantitative estimation of necrosis patches/spots frequency (NF):	Discrete 1 to 9
SI; I%I	Leaf degree of resistance to *P. viticola*	Susceptibility index (SI)/Infection index (I%I): calculation according to Townsend and Heuberger (1943)	0–100%
ΣOIV 452-1	Leaf degree of resistance to *P. viticola*	Sum of daily visual semi-quantitative estimation of DM infection	Cumulative OIV 452-1 scores
AUDPC	Leaf degree of resistance to *P. viticola* or *E. necator*	Calculation according to Jeger and Viljanen-Rollinson (2001)	According to the scale of single time point assessment

Abbreviations: AUDPC, Area Under Disease Pressure Curve; i, inverted scale; OIV, Organisation Internationale de la Vigne et du Vin.

**Table 7:** Resistance phenotyping strategies utilized in the laboratory. The main elements and possible sources of variation of phenotyping protocols adopted in the main mapping studies are reported for each *Rpv* and *Ren/Run* locus.

**Locus**	**Plant material**			**Pathogen inoculum**			**Experimental conditions**	**Resistance variables ID**	**Experimental design**			**References**
	**Obs. unit**	**Origin**	**Leaves age**	**Origin**	**Way of inoc**.	**Conc**.			**Unit NB**	**Time of obs**.	**Replicates**	
*Rpv2*	MD	MD	MD	MD	MD	MD	MD	MD	MD	7 dpi	MD	Wiedemann-Merdinoglu et al., 2006
*Rpv3.1; Rpv7; Rpv11*	Leaf disks	Greenhouse	4-5th node	Plants in CC	Spray	1.5 x 10^5^ sporangia/ml	21°C; 16–8 h light-dark	OIV 452-1; DI; AUDPC; SD; NI; SCCv; SZ; MYC	Up to 16	Between 2 and 6 dpi	2	Bellin et al., 2009
	Leaf disks	Field	4-5th node	Plants in CC	Soaking	5 x 10^4^ sporangia/ml	21°C; 16–8 h light-dark		16	Between 4 and 6 dpi	MD	
*Rpv3.1*	Leaf disks	Greenhouse	5-6th node	Leaves in CC	Drop	5 x 10^5^ sporangia/ml	21°C; 16–8 h light-dark	OIV 452-1	10	6 dpi	3	van Heerden et al., 2014
*Rpv3.1; Rpv3.2*	Leaf disks	Greenhouse	4-5th node	Plants in CC	Spray	1 x 10^5^ sporangia/ml	21°C; 16–8 h light-dark	OIV 452-1; NI; NF; NS; SCCv; SD; SPO; SZ	Up to 16	5 dpi	1	Zyprian et al., 2016
	Leaf disks	Greenhouse	3-4th node	Field	Drop	2 x 10^4^ sporangia/ml	25°C; 12–12 h light-dark			5 dpi	3	
*Rpv3.3*	Leaf disks	Greenhouse and growth chamber	4-5th node	Plants in CC	Spray	1 x 10^5^ sporangia/ml	24°C; 24 h dark after inoculation	OIV 452-1; DI; DS	8	4-5-6 dpi	2	Vezzulli et al., 2019
*Rpv5; Rpv6*	Leaf disks	Greenhouse	5-6th exp. leaf	Plants in CC	Spray	1 x 10^5^ sporangia/ml	21°C; 16–8 h light-dark	OIV 452-1; SCc	16	6 dpi	2	Marguerit et al., 2009
*Rpv8*	Leaf disks	Greenhouse	4-5th exp. leaf	Plants in CC	Spray	1 x 10^5^ sporangia/ml	21°C; 16–8 h light-dark	OIV 452-1; DI; NI; NS; SCc.	16	5 dpi	3	Blasi et al., 2011
*Rpv10; Rpv11*	Leaf disks	Greenhouse	3rd-4th node	Field	Drop	2 x 10^4^ sporangia/ml	25°C; 12–12 h light-dark	OIV 452-1	4	5-7 dpi	4	Schwander et al., 2012
*Rpv12*	Leaf disks	Greenhouse	4-5th node	Plants in CC	Spray	1 x 10^5^ sporangia/ml	21°C; 16–8 h light-dark	OIV 452-1; ΣOIV 452-1; NI	16	Between 3 and 8 dpi	MD	Venuti et al., 2013
*Rpv14*	Leaf disks	Field	3rd-4th node	Field	Drop	2 x 10^4^ sporangia/ml	25°C; 12–12 h light-dark	OIV 452-1i	4	5–7 dpi	2	Ochssner et al., 2016
*Rpv17; Rpv18; Rpv19; Rpv20; Rpv21*	Leaf disks	Field	5th node	Vitro	Drop	5 x 10^4^ sporangia/ml	23°C	DS; DSa; NS; NSa	8	Between 2 and 8 dpi	5	Divilov et al., 2017, 2018
*Rpv22; Rpv23; Rpv24*	Leaf disks	Greenhouse	4-5th node	Field	Drop	1 x 10^5^ sporangia/ml	21°C; 12–12 h light-dark	OIV 452-1; SD; Dsa	16	5-6-7 dpi	3	Fu et al., 2020
*Rpv25; Rpv26*	Leaf disks	Greenhouse	5-6th node	Field	Drop	1 x 10^5^ sporangia/ml	20°C; 16–8 h light-dark	SDi; SI	10	7 dpi	5	Lin et al., 2019
*Rpv27*	Leaf disks	Field	3rd-shiny half-size of matured leaf	Vitro	Spray	1 x 10^5^ sporangia/ml	14–10 h light-dark	DS	8	Daily for 8 dpi	4	Sapkota et al., 2019
*Rpv28*	Leaf disks	Greenhouse	3rd-4th apical insertion	Leaves in CC	Spray	7 x 10^4^ sporangia/ml	21°C; 19–5 h light-dark	OIV 452-1	8	7 dpi	1	Bhattarai et al., 2021
*Rpv29; Rpv30; Rpv31*	Leaf disks	Greenhouse	3rd-5th node	MD	Spray	5 x 10^4^ sporangia/ml	22°C	DS; I%I	3	10 dpi	3	Sargolzaei et al., 2020
*Ren1;* *Ren2*	Leaf disks	Field	3rd node	Leaves *in vitro*	Spray	1 x 10^5^ conidia/ml	23°C; 12–12 h light-dark	DS; DI; HZ; HT	4	8-9 dpi		Cadle-Davidson et al., 2016
*Ren1.2*	Leaves	Greenhouse	3rd-4th node	Leaves *in vitro*	Settling tower	MD	MD	DS	4	14-15 dpi	1	Riaz et al., 2020
*Ren1.2*	Leaf disks	Greenhouse	2nd-4th shiny and exp. leaf	Leaves *in vitro*	Settling tower	6–8 conidia/mm^2^	23°C; 16–8 h light-dark	MYC; SPO; SCc; NI; AUDPC	1	Between 2 and 11 dpi	Up to 3	Possamai et al., 2021
*Ren3; Ren9*	Leaves	Field	3rd-4th node	Plants in CC	Brush contact	MD	MD	MYC; SPO; NI	3	Between 7 and 9 dpi	2	Zendler et al., 2017
	Leaf disks	Greenhouse	MD	Vitro	Spray	1-2 x 10^5^ conidia/ml	23°C; 12– 12h light-dark	DS; NS	4	4-6 dpi	MD	Zendler et al., 2021a
*Ren4*	Leaves	Greenhouse	4th exp. leaf	Field	Spray	5 x 10^4^ conidia/ml	20°C; 12–12 h light-dark	DS	8	21 dpi	1	Ramming et al., 2011
*Ren5*	Leaves	Greenhouse	2nd-3rd exp. leaf	Vitro	Settling tower	MD	25°C; 18-6 h light-dark	MYC; SPO	2	3-4-5-6-7 dpi	2	Blanc et al., 2012
*Ren6; Ren7*	Leaves	Greenhouse	3rd-4th exp. leaf	Vitro	Settling tower	2.18 conidia/mm^2^	MD	DS; qPCR	4	14 dpi	1	Pap et al., 2016
*Run1*	Leaf disks	MD	MD	Vitro	Settling tower	MD	MD	DI	3	10 dpi	1	Pauquet et al., 2001

Abbreviations: CC, control conditions; dpi, days-post-inoculation; exp, expanded; inoc, inoculation; MD, missing data; No., number; Obs, observation; Resistance variable ID: see Table 6.

### Plant material management

For the bioassays performed in the laboratory, young leaves are collected from plants growing in optimal conditions either in the greenhouse (Blanc et al., 2012; Vezzulli et al., 2019; Possamai et al., 2021) or vineyard (Bellin et al., 2009; Ochssner et al., 2016; Divilov et al., 2017; Sapkota et al., 2019). Leaves are usually collected according to their position along the shoot to avoid ontogenic resistance (Merry et al., 2013; Calonnec et al., 2018, 2021) (Table 7). The maximal susceptibility to PM infection is conserved up to the fourth to fifth unfolded leaf on cuttings (Merry et al., 2013) and up to the second to third leaf on shoots in the vineyard (Barba et al., 2015; Calonnec et al., 2018). Some authors suggested that shine is also an important factor to identify susceptible leaves (Sapkota et al., 2019), particularly for PM resistance evaluations (Feechan et al., 2011; Cadle-Davidson et al., 2016; Possamai et al., 2021).

At the beginning of phenotyping experiments, particularly for *E. necator* resistance evaluations, a well-established practice is leaf disinfection and rinsing (Ramming et al., 2011; van Heerden et al., 2014; Pap et al., 2016; Divilov et al., 2017; Vezzulli et al., 2018; Sapkota et al., 2019; Bhattarai et al., 2021; Karn et al., 2021; Possamai et al., 2021; Zendler et al., 2021a). This avoids contamination when plant material originates in the vineyard and for long-lasting experiments.

Between 8 and 16 leaf disks are usually excised for DM resistance evaluations (Bellin et al., 2009; Divilov et al., 2017; Vezzulli et al., 2019), while for PM resistance evaluations, up to eight whole leaves (Ramming et al., 2011; Blanc et al., 2012; Pap et al., 2016) or leaf disks are assessed (Pauquet et al., 2001; Karn et al., 2021; Possamai et al., 2021; Zendler et al., 2021a).

### Inoculum and infection management

*P. viticola* and *E. necator* inocula can either be collected as a natural population in the vineyard (Ramming et al., 2011; Schwander et al., 2012; Lin et al., 2019; Fu et al., 2020), or produced under controlled conditions on potted plants, for *P. viticola* (Bellin et al., 2009; Vezzulli et al., 2019), or on *in vitro* leaves for both pathogens (Blanc et al., 2012; Pap et al., 2016; Divilov et al., 2017; Sapkota et al., 2019; Possamai et al., 2021). Controlled inocula may originate as a mixture of isolates (Hoffmann et al., 2008; Bellin et al., 2009; Zyprian et al., 2016), or monosporangia/monoconidial cultures (Bellin et al., 2009; Teh et al., 2017; Possamai et al., 2021) (Table 7). In mapping studies, isolates are usually characterized by the pathogen origin (Bellin et al., 2009; Barba et al., 2015; Sargolzaei et al., 2020) and a few defined isolates (Bellin et al., 2009; Venuti et al., 2013). Leaves with *P. viticola* sporulation, or sporulated lesions, can be conserved by freezing (Delmotte et al., 2014), but the aggressiveness of the pathogen inoculum requires an infection cycle on leaves before resistance phenotyping. In contrast, *E. necator* isolates need to be maintained on green tissue (Miazzi et al., 1997; Gao et al., 2016).

For DM leaf disc bioassays, the pathogen inoculum is prepared by soaking leaves showing fresh *P. viticola* sporulation in water (Bellin et al., 2009; Divilov et al., 2017). Then, inoculations are performed by spraying the solution on the tissues (Bellin et al., 2009; Sapkota et al., 2019; Vezzulli et al., 2019; Sargolzaei et al., 2020; Bhattarai et al., 2021) or by the application of solution drops (10–50 μl) on the abaxial surface of the leaves (Schwander et al., 2012; Divilov et al., 2017; Lin et al., 2019; Fu et al., 2020). Experiments used a concentration of the spore solution ranging from 2 × 10^4^ to 5 × 10^5^ sporangia per ml (Table 7). *Plasmopara viticola* droplets remain on leaves for up to 24 h and need to be removed to avoid tissue rotting (van Heerden et al., 2014; Zyprian et al., 2016).

*Erysiphe necator* is inoculated on *in vitro* leaves by spraying conidia suspensions (Ramming et al., 2011; Cadle-Davidson et al., 2016; Karn et al., 2021; Zendler et al., 2021a) or using settling towers (Pauquet et al., 2001; Blanc et al., 2012; Pap et al., 2016; Possamai et al., 2021). Experiments used PM pressure ranging from 5 × 10^4^ to 2 × 10^5^ conidia per ml for spraying, and from 2 to 8 conidia per mm^2^ for settling towers (Table 7).

Petri dishes containing leaves or leaf disks are usually inoculated at the sampling date and incubated according to *P. viticola* and *E. necator* optimal growth and sporulation conditions between 20 and 25°C and with high relative humidity. Photoperiods vary between 12 and 19-h light (Schwander et al., 2012; Bhattarai et al., 2021) (Table 7). *P. viticola* infection is usually evaluated between 5 and 7 dpi (Bellin et al., 2009; Schwander et al., 2012; Lin et al., 2019; Fu et al., 2020; Bhattarai et al., 2021), while *E. necator* infection is assessed between 3 and 14 dpi (Blanc et al., 2012; Pap et al., 2016; Possamai et al., 2021).

### Assessment of resistance variables from *in vitro* bioassays

In the laboratory, resistance rating methods are very diverse (Tables 6, 7). As commonly practiced for the other environments, DM and PM sporulation is scored for incidence (Pauquet et al., 2001; Bellin et al., 2009; Blasi et al., 2011; Vezzulli et al., 2019) and severity using different visual semi-quantitative scales (Divilov et al., 2017; Sapkota et al., 2019; Vezzulli et al., 2019), such as the OIV 452-1 and 455-1 descriptors (OIV, 2009). *In vitro* bioassays facilitate the use of machine vision analyses (Divilov et al., 2017; Fu et al., 2020). Phenotyping in the laboratory is often enriched by the collection of additional variables obtained by stereomicroscopes and microscopes. Some examples are: DM density of sporangiophores (Bellin et al., 2009; Zyprian et al., 2016; Fu et al., 2020), DM mesophyll colonization (Bellin et al., 2009), PM mycelium growth (Blanc et al., 2012; Possamai et al., 2021), dimension of DM sporangia (Zyprian et al., 2016), count of PM hyphal transects (number of hyphae crossed by a line traversing the leaf disc; Cadle-Davidson et al., 2016; Karn et al., 2021), and spore production (Zyprian et al., 2016; Possamai et al., 2021) (Tables 6, 7). *In vitro* phenotyping is also often enriched by the calculation of infection indexes (Townsend and Heuberger, 1943; Lin et al., 2019; Sargolzaei et al., 2020).

Plant necrosis is assessed either qualitatively (Bellin et al., 2009; Zyprian et al., 2016; Possamai et al., 2021) or quantitatively for its frequency and extension (Blasi et al., 2011; Zyprian et al., 2016; Divilov et al., 2017; Zendler et al., 2021a), in particular for *P. viticola* infections (Tables 6, 7).

Time series ratings are frequently recorded in the laboratory (Bellin et al., 2009; Venuti et al., 2013; Divilov et al., 2017; Sapkota et al., 2019; Vezzulli et al., 2019; Possamai et al., 2021) (Table 6), allowing several comparisons among phenotyping data and providing the opportunity to obtain new variables that consider the complete infection process, such as the Area-Under-Disease-Pressure-Curve (AUDPC; Jeger and Viljanen-Rollinson, 2001).

### QTL identification through *in vitro* bioassays

*In vitro* bioassays detected all the resistance QTL loci identified in the vineyard, such as *Rpv3.1* (Bellin et al., 2009; Zyprian et al., 2016), *Rpv12* (Venuti et al., 2013), *Rpv14* (Ochssner et al., 2016), *Rpv27* (Sapkota et al., 2019), and *Ren3/Ren9* (Zendler et al., 2017), or in the greenhouse, such as *Rpv3.3* (Vezzulli et al., 2019), *Rpv28* (Bhattarai et al., 2021), and *Ren1.2* (Riaz et al., 2020; Possamai et al., 2021). The QTL loci *Ren1* (Hoffmann et al., 2008; Cadle-Davidson et al., 2016), *Ren4* (Ramming et al., 2011), *Ren6, Ren7* (Pap et al., 2016), and *Run1* (Pauquet et al., 2001) were detected in all the phenotyping environments (Table 6). *In vitro* assays provided both QTL significances close (Bellin et al., 2009; Pap et al., 2016; Vezzulli et al., 2019; Riaz et al., 2020) or far (e.g., Bhattarai et al., 2021) from those identified in other phenotyping environments.

Through phenotyping *in vitro*, several other resistance loci were mapped, in particular for *P. viticola* (Table 6), confirming the suitability of such a strategy to assess large numbers of accessions. QTL with large significant effects and/or stable results between experiments were *Rpv5, Rpv6* (Marguerit et al., 2009), *Rpv8* (Blasi et al., 2011), *Rpv10* (Schwander et al., 2012), *Rpv22, Rpv23, Rpv*24 (Fu et al., 2020), and *Ren5* (Blanc et al., 2012). In the study of *Rpv17, Rpv18, Rpv19, Rpv20*, and *Rpv21*, the proposed loci were not identified in all the mapping populations and experiments. Additionally, they were mapped together with several minor loci (Divilov et al., 2018). The *Rpv25* and *Rpv26* loci were not consistently identified and were detected together with other unstable QTLs (Lin et al., 2019). Finally, the *Rpv*29, *Rpv30*, and *Rpv*31 loci (Sargolzaei et al., 2020) segregated only in a few seedlings of the mapping population and accessions studied, which showed a large variability in infection rate.

*In vitro* bioassays suggest a certain variation of phenotyping in relation to the physiological state of the plant and aggressiveness of the pathogen (Sargolzaei et al., 2020; Possamai et al., 2021). Therefore, unoptimized and unstandardized practices are probably the cause of unstable QTL detection and significant differences in comparison with the other phenotyping environments. However, some authors suggested that minor QTL are easily detected under optimal laboratory conditions (Riaz et al., 2011; Teh et al., 2017).

The visual evaluation of disease incidence and severity, and the quantification of pathogen structures (sporangia or conidia count) produced the most significant QTL identification (Bellin et al., 2009; Marguerit et al., 2009; Blasi et al., 2011; Zyprian et al., 2016; Vezzulli et al., 2019; Possamai et al., 2021). For instance, mycelium growth and sporulation scores were the best variables in characterizing QTLs of total (Blanc et al., 2012; Pap et al., 2016) and partial (Possamai et al., 2021) resistance to *E. necator*. Objective DM severity carried out by machine vision analysis (Divilov et al., 2017; Fu et al., 2020), sporulation quantified by cell counters (Bellin et al., 2009; Blasi et al., 2011) and severity of *E. necator* measured by hyphal-transect (Cadle-Davidson et al., 2016; Karn et al., 2021) and qPCR (Pap et al., 2016) also identified major QTLs with high significance. Other variables, such as *P. viticola* mesophyll colonization (Bellin et al., 2009) and sporangia size (Zyprian et al., 2016), provided interesting biological information but were less effective in identifying resistance QTLs.

Plant necrosis provided resistance QTL collocating with those identified by sporulation data for *Rpv3.1* (Welter et al., 2007), *Rpv8* (Blasi et al., 2011), and *Ren1.2* (Possamai et al., 2021), but also revealed new loci such as *Rpv3.2* (Zyprian et al., 2016) and *Rpv17* (Divilov et al., 2018).

The assessment of DM and PM over time showed that the QTL significance changed with disease progression (Bellin et al., 2009; Cadle-Davidson et al., 2016; Pap et al., 2016; Zendler et al., 2017; Possamai et al., 2021) and that cumulative infection indexes captured more phenotypic variance than single time point ratings, enhancing the QTL detection (Bellin et al., 2009; Teh et al., 2017; Karn et al., 2021; Possamai et al., 2021).

## Efforts toward a standardization of main factors influencing phenotyping data

Mapping for resistance aims to characterizing genetic sources useful for grape breeding. However, the explained phenotypic variance of QTL varies according to the collected phenotypes (Bellin et al., 2009; Pap et al., 2016; Zyprian et al., 2016; Karn et al., 2021; Possamai et al., 2021), stressing the impact of phenotyping for studying the genetic basis of resistance traits.

Our review encountered a wide variety of phenotyping protocols, highlighting the main factors with a direct effect on the significance and reproducibility of results. Resistance phenotypes depend on the biological (plants and pathogens) and environmental components involved in the experiments and their interactions, the technical difficulties in managing and standardizing the resistance evaluations, and the consequential costs and time associated with the phenotyping practices. Objective and quantitative measures of grapevine mildew resistance using new tools, but also the harmonization of the procedure according to Minimum Information About a Plant Phenotyping Experiment (MIAPPE) would improve future phenotyping protocols.

### Environments, plant material, and pathogen inocula

Phenotyping in the vineyard yield resistance data at the whole plant level during natural polycyclic infections and under unstable epidemic pressure. Some studies suggested that the leaves may be a proxy of whole plant resistance (Welter et al., 2007; Ramming et al., 2011; Pap et al., 2016; Zyprian et al., 2016; Karn et al., 2021). However, other studies suggested limited correlation between resistance of grapevine leaves and bunches (Calonnec et al., 2013; Buonassisi et al., 2018; Vezzulli et al., 2018). This may be due to either the presence of factors that influence the susceptibility of the organs or a possible low reliability of some resistance data. For these reasons, resistance should be observed on different organs, at least leaves and bunches. Furthermore, to standardize phenotyping results, the time of plant organ scoring should be defined according to the grapevine phenological phases (Lorenz et al., 1995; OIV, 2009). For disease dynamic, epidemic pressures could be estimated by assessing control plants (Riaz et al., 2011; Ochssner et al., 2016; Zyprian et al., 2016), or by capturing and quantifying pathogen airborne spores (Mahaffee and Stoll, 2016; Thiessen et al., 2018; Brischetto et al., 2020). Finally, pedoclimatic data should be recorded to better understand the resistance phenotyping results and QTL analyses (Zyprian et al., 2016; Zendler et al., 2017, 2021a).

Phenotyping under natural conditions of infection in the greenhouse has similar characteristics to phenotyping in the vineyard but with more consistent environmental conditions. Plants produced in the greenhouse for subsequent greenhouse or *in vitro* experiments allow a better follow-up of plant growth and juvenility of the leaves (Welter et al., 2007; Zyprian et al., 2016). The growing degree-day and/or the leaf position on the shoots of the leaf collected can be helpful to record the stage of the plant material used in phenotyping. Pathogen inoculations could be also standardized using artificial inoculation of isolates maintained under control conditions. However, the management of plant growth and infection in the greenhouse may result in expensive and complex practices in terms of space and time, limiting bioassay replicates. Probably for these reasons, only a limited number of experiments are carried out in the greenhouse.

*In vitro* bioassays do not affect the original plants and can be performed at different periods and in multiple replicates with several experimental units during the vegetative season. To date, pathogen isolates used in mapping studies have been poorly described. Therefore, it would be advantageous to standardize pathogen inocula *in vitro* using a set of isolates characterized for their virulence and aggressiveness. *In vitro* evaluations are effective in describing the biological and genetic resistance components during one pathogen cycle, but the bioassays do not consider possible derivative effects of the polycyclic behavior of the disease (Bove et al., 2019). Furthermore, plants are tested in extremely favorable conditions for the diseases, leading to a possible underestimation of the resistance in comparison with the vineyard (Calonnec et al., 2013). Therefore, the extreme diverse experimental conditions may explain the limited correlation between resistance sometimes observed on detached leaves or leaf disks and bunches in the vineyard (Calonnec et al., 2013; Zyprian et al., 2016; Buonassisi et al., 2018; Vezzulli et al., 2018; Bove et al., 2019). For all these reasons, resistant plants with new identified QTL should be assessed also under vineyard conditions.

Regardless of the phenotyping environment, the use of a range of control genotypes (with known resistance loci correlated to various resistance levels) growing and rated in the same environment and experimental design as the candidate plants to be assessed, would validate the phenotyping practices (plant material growth and sampling, pathogen inoculum preparation, and distribution and infection environmental conditions). Finally, due to the possible pathogen genetic variability, the identified resistance QTL should be checked in multi-year and multi-time evaluations, and in different vineyard areas before their introduction into breeding programs (Bellin et al., 2009; Teh et al., 2017; Karn et al., 2021; Possamai et al., 2021).

### Resistance variables

The different variables used to score *P. viticola* and *E. necator* symptoms show significant correlations between them and usually locate the main QTL at the same genomic positions (Bellin et al., 2009; Cadle-Davidson et al., 2016; Pap et al., 2016; Zyprian et al., 2016; Karn et al., 2021; Possamai et al., 2021). Sporulating *P. viticola* and *E*. *necator* is primarily utilized to characterize grapevine resistance because it is macroscopic and appraisable in all phenotyping environments (Bellin et al., 2009; Venuti et al., 2013; Pap et al., 2016; Zyprian et al., 2016; Teh et al., 2017; Vezzulli et al., 2018; Fu et al., 2020; Sargolzaei et al., 2020; Özer et al., 2021). However, different studies suggest that necrosis or cell death (Pitsili et al., 2020) should be also assessed because the plant response may be differentially expressed according to plant–pathogen interactions (Zyprian et al., 2016; Gómez-Zeledón et al., 2017; Divilov et al., 2018; Possamai et al., 2020).

The official visual semi-quantitative scales, such as those of OIV or IPGRI, are considered subjective and error-prone (Poland and Nelson, 2011). However, annual training of observers in visual estimation of disease symptoms could reduce possible biases. To further reduce the possibility and effects of human errors and to improve the biological description of resistance and QTL identification, it is advisable that resistance assessments should be performed by several people, on multiple biological replicates and in multiple bioassays. The disease severity rating method may be an alternative to the previous scales, but the assessment of the percentage of infected surface may also be subjective. The disease incidence is probably the easiest method to assess resistance but fails to record complete information about quantitative phenotypes.

### Tools and strategies for improving grapevine resistance phenotyping objectivity and automation

In resistance mapping studies, despite the numerous experiments and large-scale samples, phenotyping is generally conducted by traditional visual rating of disease symptoms with some exceptions (Pap et al., 2016; Divilov et al., 2017, 2018; Fu et al., 2020). Other studies showed that metabolomes such as stilbenoids in grapevines (Malacarne et al., 2011; Chitarrini et al., 2017) and lipids in pathogens (Negrel et al., 2018) can be studied to characterize DM and PM infections. Alternatively, grapevine pathogens can also be detected and quantified by molecular techniques such as qPCR and transcriptional analyses (Valsesia et al., 2005; Dufour et al., 2011; Malacarne et al., 2011; Hariharan and Prasannath, 2021). Different metabolic responses to *P. viticola* have already been associated with resistance loci (Eisenmann et al., 2019; Chitarrini et al., 2020; Ciubotaru et al., 2021) and could provide guidelines to define new resistance variables. However, these methods do not allow monitoring of disease progression.

Assessment of grapevine tissue, using spectrometers and spectral imaging sensors, relies on non-destructive measurements capable of early detection of pathogen infection, tracking of infection progression, and differentiation of responses mediated by resistance loci (Cséfalvay et al., 2009; Peressotti et al., 2011; Oerke et al., 2016; Štambuk et al., 2021). Nowadays, machine vision approaches can be highly automated in image capture, and their analysis can use advanced recognition algorithms (Hamuda et al., 2017) and convolutional neural networks (CNNs) (Rawat and Wang, 2017). These elements increase the machine vision throughput and performance that rival human observers in grapevine disease detection and classification (Bierman et al., 2019; Liu et al., 2020; Zendler et al., 2021b). Disease severity assessed by machine vision appears to be time and cost-effective (Divilov et al., 2017), but presents specific challenges, such as droplet dispersion (Fu et al., 2020), leaf morphology (Divilov et al., 2017; Fu et al., 2020), and low pathogen structure (*E. necator* hyphae) resolution (Bierman et al., 2019). Machine vision probably represents one of the most interesting opportunities to produce objective and quantitative measures of grapevine resistance.

### Toward minimum information about a plant phenotyping experiment

The lack of common standards for grapevine resistance phenotyping experiments (plant material and pathogen inoculum, collection and storage) may hamper the correct interpretation of mapping studies and their application to new phenotyping and genetic investigations. This lack of standards is the result of a large variability of protocols but also of the dependence of the genetic trait on the environment and phenotyping conditions (Krajewski et al., 2015).

The Minimum Information About a Plant Phenotyping Experiment (MIAPPE) was proposed to harmonize phenotypic observations and develop precise and repeatable protocols (Krajewski et al., 2015; Cwiek-Kupczyńska et al., 2016; Papoutsoglou et al., 2020). These authors inventoried the attributes that are necessary for a useful description of a plant phenotyping experiment and an optimal use and re-use of the protocol and data produced. The MIAPPE (www.miappe.org) checklist consists of attributes that can be classified within the following eight categories: (1) general metadata (dataset identifier, objectives of the experiment and authors); (2) location and timing of the experiment; (3) biosource (plant material identification, pathogen); (4) environmental descriptors (for vineyard, greenhouse and environment chamber) such as aerial conditions, light, fertilizing and watering; (5) treatments (environmental properties, artificial treatments); (6) experimental design (experimental units could be a single plant, plot and groups of plants); (7) samples collection, processing and management protocols; and (8) observed variables described by three basic attributes: trait name, measurement method, and notation scale. Furthermore, authors relied on annotations that agree with publicly available ontologies using proper dataset formats (ISA-Tab; Rocca-Serra et al., 2010) to ensure the understanding and interoperability of the experiments (Krajewski et al., 2015; Cwiek-Kupczyńska et al., 2016). In particular, observed variable attributes were formalized through the Crop Ontology (CO; Shrestha et al., 2012; www.cropontology.org). For grapevine resistance phenotyping, this approach is still new and guidelines are not available yet. Such guidelines need be developed to implement best practices, improve the quality, availability, and usefulness of the data and to associate metadata describing the experiments. The implementation of such standards would facilitate the harmonization of data and metadata resources in a shared format (Savoi et al., 2021).

## Conclusions

During the past 20 years, effective protocols have been implemented in all the environments of phenotyping (vineyard, greenhouse and laboratory conditions) for the identification of several DM and PM resistance *Rpv* and *Ren/Run* loci, respectively. Experimental conditions in the vineyard are variable and mostly uncontrolled (plant growth, pathogen inoculum, and climate). Additionally, they affect the degree of DM and PM infections, and significance of the QTL analyses. Phenotyping in more controlled environments such as the greenhouse or laboratory is often more effective than in the vineyard because it allows the production of reproducible results that increase the reliability of QTL identification. However, controlled environments generally provide only partial information on the resistance of the genotypes studied, such as leaf resistance under conditions of monocyclic pathogen development. Thus, with an aim of resistance durability, grapevine genotypes identified as resistant to DM and PM under these conditions should be evaluated in the vineyard where natural and diverse pathogen populations occur. Finally, in the search for new resistance loci in grapevine, future phenotyping experiments should follow MIAPPE guidelines to standardize protocols and increase the reliability and reproducibility of QTL mapping results, regardless of the phenotyping environment. Meanwhile, phenomics could provide new tools to increase grapevine-resistance assessment throughput and accuracy.

## Author contributions

TP and SW-M conceived the work, performed the literature search, analyzed the data, drafted, and edited the manuscript. Both authors contributed to the article and approved the submitted version.

## Conflict of interest

The authors declare that the research was conducted in the absence of any commercial or financial relationships that could be construed as a potential conflict of interest.

## Publisher's note

All claims expressed in this article are solely those of the authors and do not necessarily represent those of their affiliated organizations, or those of the publisher, the editors and the reviewers. Any product that may be evaluated in this article, or claim that may be made by its manufacturer, is not guaranteed or endorsed by the publisher.

## Abbreviations

BC: Backcross
DM: Downy Mildew
dpi: Days Post Infection/Inoculation
GWAS: Genome-Wide Association Study
IPGRI: International Plant Genetic Resources Institute
LG: Linkage Group
MIAPPE: Minimum Information About a Plant Phenotyping Experiment
MD: Missing Data
OIV: Organization Internationale de la Vigne et du Vin
PM: Powdery Mildew
QTL: Quantitative Trait Locus
RH: Relative Humidity
*Rpv*: Resistance to *Plasmopara Viticola*
*Ren*: Resistance to *Erysiphe Necator*
*Run*: Resistance to *Uncinula Necator*
UPOV: Union for the Protection of New Varieties of Plants.
